# Subsidence Characteristics in North Anhui Coal Mining Areas Using Space–Air–Ground Collaborative Observations

**DOI:** 10.3390/s24123869

**Published:** 2024-06-14

**Authors:** Li’ao Quan, Shuanggen Jin, Jianxin Zhang, Junyun Chen, Junjun He

**Affiliations:** 1 Shanghai Astronomical Observatory, Chinese Academy of Sciences, Shanghai 200030, China; laquan@shao.ac.cn; 2 School of Astronomy and Space Science, University of Chinese Academy of Sciences, Beijing 100049, China; 3 The Fourth Surveying and Mapping Institute of Anhui Province, Hefei 230031, China; 4 School of Surveying and Land Information Engineering, Henan Polytechnic University, Jiaozuo 454003, China

**Keywords:** coal mining subsidence, InSAR, UAV LiDAR, land use, collaborative observations

## Abstract

To fully comprehend the patterns of land and ecological damage caused by coal mining subsidence, and to scientifically carry out ecological mine restoration and management, it is urgent to accurately grasp the information of coal mining, particularly in complex coaling areas, such as North Anhui, China. In this paper, a space–air–ground collaborative monitoring system was constructed for coal mining areas based on multi-source remote sensing data and subsidence characteristics of coaling areas were investigated in North Anhui. It was found that from 2019 to 2022, 16 new coal mining subsidence areas were found in northern Anhui, with the total area increasing by 8.1%. In terms of land use, water areas were increased by 101.9 km^2^ from 2012 to 2022, cultivated land was decreased by 99.3 km^2^, and residence land was decreased by 11.8 km^2^. The depth of land subsidence in the subsidence areas is divided into 307.9 km^2^ of light subsidence areas with a subsidence depth of less than 500 mm; 161.8 km^2^ of medium subsidence areas with a subsidence depth between 500 mm and 1500 mm; and 281.2 km^2^ of heavy subsidence areas with a subsidence depth greater than 1500 mm. The total subsidence governance area is 191.2 km^2^, accounting for 26.5% of the total subsidence area. From the perspective of prefecture-level cities, the governance rate reaches 51.3% in Huaibei, 10.1% in Huainan, and 13.6% in Fuyang. The total reclamation area is 68.8 km^2^, accounting for 34.5% of the subsidence governance area. At present, 276.1 km^2^ within the subsidence area has reached stable subsidence conditions, mainly distributed in the Huaibei mining area, which accounts for about 60% of the total stable subsidence area.

## 1. Introduction

As an important basic energy source in China, coal resources have played an irreplaceable role in the development of the national economy [1]. Long-term coal mining has caused large-scale subsidence of the land surface, which causes ecological environment damage, loss of cultivated land, and damage of houses, etc. [2] Therefore, it is imperative to promote comprehensive management of coal mining subsidence areas and implement the idea of ecological civilization. It is a practical need to improve people’s livelihood and promote high-quality development.

Since the subsidence has a large effect area with wide distribution and involving a large population, its governance requires huge investment. Systematic and scientific investigation and monitoring work should be carried out during the governance process [3]. On the basis of a full investigation and research, based on the work orientation of “two services, two supports” of China’s surveying, mapping and geoinformation industry, the current needs of the natural resource management department for monitoring coal mining subsidence areas are as following: (1) Accurately measure coal mining subsidence areas scope and clarify the affected areas on the surface. (2) Accurately grasp the changes in land use in recent years within the coal mining subsidence areas to serve as the formulation of territorial spatial planning. (3) Implement the protection of cultivated land and find out the degree of damage and impact of cultivated land for the next step, and provide suggestions for land reclamation. (4) Know the stable subsidence area within the subsidence area, and accurately obtain this range to formulate scientific and standardized governance models [4].

The range of surface subsidence caused by coal mining needs to be determined by monitoring changes in surface subsidence. The traditional measurement method is mainly by leveling, and leveling points are laid out within the affected range to ensure a certain monitoring frequency [5], which has a limited number of monitoring points and low spatial coverage. As CORS (Continuous Operating Reference Station) can provide higher positioning accuracy, GNSS has been used to measure more monitoring points, but it is still difficult to cover the entire area [6]. InSAR technology can provide planar and sustainable surface deformation monitoring, and has gradually been widely used in subsidence monitoring in subsidence areas [7,8]. Then, DS-InSAR [9], PS-InSAR [10], SBAS-InSAR [11], and other technologies have also been studied and applied. In recent years, multi-phase DEM data of subsidence areas through Unmanned Aerial Vehicle (UAV) LiDAR technology is also a hot topic to monitor subsidence changes [12].

Coal mining brings strong human interference, and the land use type in the coal mining subsidence area changes drastically. The land use type in the coal mining subsidence area has its own unique characteristics. Some scholars have constructed a surface cover monitoring method for small areas of coal mining subsidence, and an index system to study land use and land cover changes in coal mining subsidence areas [13]. According to the land use types of concern, such as cultivated land protection, water area development, and residential area relocation, corresponding with remote sensing image recognition and land use type change mechanism, a number of analyses were carried out [14,15]. With the creation and evolution of subsidence areas, the spatiotemporal distribution and simulation of land use in subsidence areas can provide policy suggestions for future land planning in subsidence areas [16].

The scientific monitoring of subsidence areas can provide data support for their governance. The investigation of the governance situation mainly relies on on-site verification, using conventional measuring equipment and mobile tablets to investigate governance and reclamation conditions [17]. For better governance, it is necessary to understand the land damage situation in the subsidence area and classify different degrees of damage [18]. The corresponding governance model should be scientifically selected, and stable subsidence areas should be governed as early as possible. Therefore, the scope division of stable subsidence areas is also a hot topic

As monitoring data and techniques become more and more abundant, coordinated space–air–ground observations can better meet the monitoring needs of natural resources. Pollution monitoring of drinking water sources [19,20], geological disasters [21,22], floods [23,24,25], etc., increasingly require space–air–ground collaborative monitoring. From the perspective of natural resource management needs [26], a systematic multi-source data survey and monitoring system has not been formed so far. Du and Yang [27] have constructed survey and monitoring systems for coal mining subsidence areas in Shanxi Province in terms of monitoring technology and data analysis, but it is not comprehensive enough. In summary, according to the specific needs of the natural resource management department, this article aims at the following works: (1) The subsidence changes in the coal mining subsidence area are monitored by InSAR technology in northern Anhui from 2019 to 2022, and the range of stable subsidence areas is extracted; (2) Using UAV LiDAR and unmanned surveying ship measurement technology, combining with high-precision historical DEM data, the range of different subsidence depths in the coal mining subsidence area in northern Anhui is delineated from 2015 to 2022; (3) The land use changes in the coal mining subsidence area from 2012 to 2022 are analyzed by collecting land use data in the subsidence area, high-resolution remote sensing satellites images and field surveys. (4) By combining historical monitoring data, ecological restoration, and other data in the subsidence area, the governance situation of the subsidence area since 2019 is analyzed; (5) Based on multi-source remote sensing data, a space–air–ground collaborative monitoring system for the coal mining subsidence area in northern Anhui is established.

## 2. Methods

**Figure 1:**
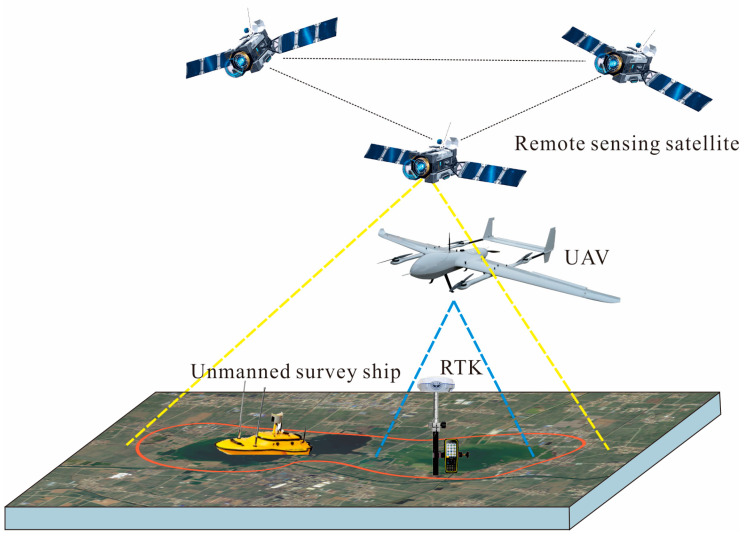
Space–air–ground collaborative observation in coal mining subsidence areas.

The space–air–ground collaborative observations of coal mining subsidence areas mainly contain satellite remote sensing, UAV LiDAR, unmanned survey ship, and GNSS-RTK technology (Figure 1), which can comprehensively monitor and grasp the area changes, land use changes, and governance status in coal mining subsidence areas.

The space–air–ground collaborative monitoring system utilizes the complementary advantages of different observation technologies to obtain observation data and field survey data at different spatial and temporal resolutions [28].

### 2.1. Optical Remote Sensing Technology

**Figure 2:**
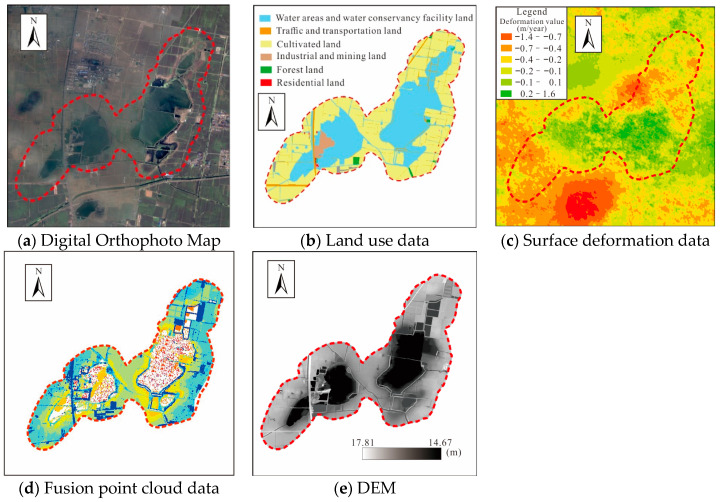
Data obtained by different technical methods (Huaibei wugou coal mine).

Satellite remote sensing technology is used to conduct large-scale, continuous, and high-precision observations of natural resource elements, which plays a role in natural resource surveys, land use change monitoring, and surface subsidence monitoring. With the rapid development of China’s domestic high-resolution earth observation technology, data sources are extremely abundant and the cost of Earth observation data has been greatly reduced [29]. High-resolution optical remote sensing images can monitor land use changes in coal mining subsidence areas, subsidence water accumulation areas, and serve as a working base map for field verification of subsidence areas. High-resolution images (Figure 2a) can be used to obtain high-precision land use data (Figure 2b).

**Figure 3:**
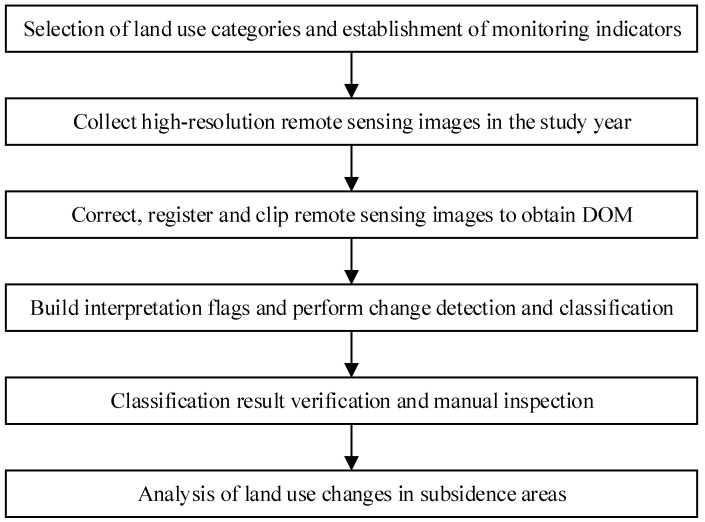
Processing of land use data in coal mining subsidence areas.

The process of extracting land use information in coal mining subsidence areas is shown in Figure 3.

### 2.2. SBAS-InSAR Technology

Different from optical remote sensing images, microwave remote sensing technology with all-weather operation capabilities can penetrate clouds, and is not easily affected by meteorological conditions and sunshine levels. SAR satellites can penetrate vegetation and have the ability to detect targets below the surface. Since Small Baseline Subset (SBAS)-InSAR can improve the coherence of interference image pairs with lower requirements for the accuracy of digital elevation model data and high sampling rate and accuracy of observation data [30], this study uses SBAS-InSAR technology to obtain surface deformation information in coal mining subsidence areas (Figure 2c).

$T_{0},T_{1}\cdots T_{N}$ are the time range of the study. First, N+1 images are obtained with covering the study period and arranged in time order to establish a spatial-temporal baseline. The differential interference pairs are combined according to the threshold requirements. The number is M. The relationship is expressedas follows [31]:

(1) $$\frac{N+1}{2}\leq M\leq \frac{N\left(N+1\right)}{2}$$

Using two SAR images at time $T_{A}$ and $T_{B}$, an interference pair ($T_{A}<T_{B}$) is formed, and the interference phase difference of the coordinate point (x,y) is expressed in the following form:

(2) $$\delta \phi_{i}\left(x,y\right)=\delta \phi_{T_{A}}\left(x,y\right)-\delta \phi_{T_{B}}\left(x,y\right)\approx \delta \phi_{i\left(disp\right)}+\delta \phi_{i\left(topo\right)}+\delta \phi_{i\left(atmo\right)}+\delta \phi_{i\left(noi\right)}$$

where $\delta \phi_{i\left(disp\right)}$, $\delta \phi_{i\left(topo\right)}$, $\delta \phi_{i\left(atmo\right)}$, and $\delta \phi_{i\left(noi\right)}$ are the deformation phase, terrain phase, atmospheric phase, and noise phase, respectively, i∈(1,…,M). The above formula is simplified to the following form with only the deformation phase:

(3) $$\delta \phi_{i}\left(x,y\right)\approx \frac{4\pi }{\lambda }\left[d_{T_{A}}\left(x,y\right)-d_{T_{B}}\left(x,y\right)\right]$$

where λ is the radar microwave wavelength, $d_{T_{A}}\left(x,y\right)$ is the deformation of point (x,y) at time $T_{A}$ relative to $T_{A}$, and $d_{T_{B}}\left(x,y\right)$ is the deformation at time $T_{B}$ relative to the initial time. Suppose IE and IS are the master image and the slave image, respectively, then the interference pairs are as follows:

(4) $$IE=\left[IE_{1},IE_{2},\cdots ,IE_{M}\right]$$

(5) $$IS=\left[IS_{1},IS_{2},\cdots ,IS_{M}\right]$$

The situation of $IE_{i}>IS_{i}$ is satisfied, where ∀i=1,2,⋯,M. Then, any interference pattern can be expressed as follows:

(6) $$\delta \phi_{i}\left(x,y\right)=\phi \left(T_{IE_{i}}\right)-\phi \left(T_{IS_{i}}\right)$$

Forming a system of equations, in which there are N unknown numbers and M equations, it is expressed in matrix form as:

(7) δϕ=Aϕ

where A is the matrix M×N. When M≥N, for the interior of the small baseline set, the least squares method is used to obtain the result; while when M<N, the singular value decomposition method is used to solve the problem of rank deficiency to obtain the minimum norm, which is the final deformation value.

### 2.3. UAV LiDAR Technology

Based on low-altitude UAV remote sensing monitoring, the refined structural information of natural resources can be obtained. UAV LiDAR technology is a combination of the advantages of UAVs and LiDAR. It can flexibly take off and land, fly at low altitudes and quickly obtain data, which can also obtain surface information through vegetation without special geographical restrictions, and then obtain high-precision three-dimensional coordinate data in real time [32,33]. UAV LiDAR measurement can obtain high-precision DEM data within the subsidence area, building height, water boundary elevation information, etc. Multi-period high-precision DEM can monitor settlement changes in the subsidence area and build a visualization model of the subsidence area. Point cloud data density should be sufficient to interpolate digital elevation model data.

The collected point cloud data (Figure 2d) are filtered by noise points, processed by overlapping flight areas, automatically classified and manually classified to obtain ground points, and finally the 2 m × 2 m DEM data are generated (Figure 2e) [34].

### 2.4. Unmanned Survey Ship Technology

As the surface sinks, large water accumulation areas will form in coal mining subsidence areas. Due to the complex internal environment of the survey area, the water areas formed are small and widely distributed. An unmanned survey ship was selected to carry out underwater terrain survey work. It can navigate autonomously in the uncertain water surface environment and complete corresponding surveying and mapping tasks [35].

**Figure 4:**
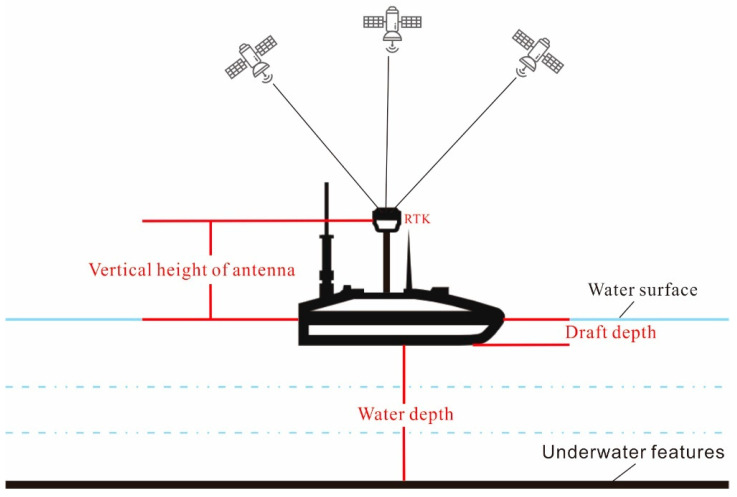
Measurement principles of unmanned survey ships.

The GNSS Real-Time Kinematic (RTK) measurement system has a differential positioning accuracy of centimeter level and can meet the bathymetric map requirements of up to 1:500. The unmanned ship is equipped with GNSS receiver and depth sounder. The high accuracy position information is obtained by GNSS RTK, the water depth information is obtained by the depth sounder, and the underwater elevation is obtained through the following formula. Finally an underwater topographic map is generated (Figure 4).

(8) $$H=H_{RTK}-h-D_{draft}-D_{water}$$

where H is underwater bottom elevation, $H_{RTK}$ is receiver antenna elevation, h is antenna vertical height, $D_{draft}$ is draft depth, and $D_{water}$ is water depth.

### 2.5. Integration of Multi-Source Data

**Figure 5:**
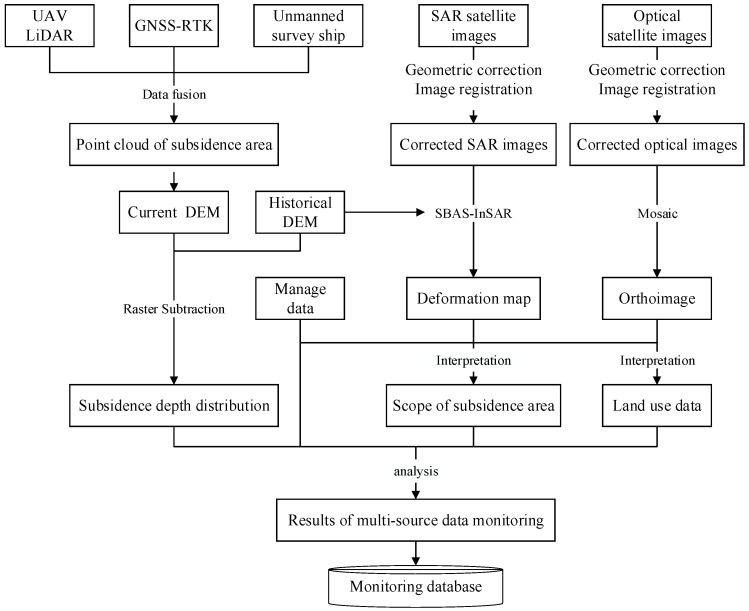
Multi-source data integration in coal mining subsidence areas.

Data fusion and mutual verification ensure the accuracy, and technologies complement each other to ensure all-round efficiency. Satellite remote sensing can monitor large-scale areas and provide macro-decisions for natural resource management. Low-altitude drone aerial surveys, unmanned survey ships, and field inspections can provide the refined monitoring data for specific business departments (Figure 5).

**Figure 6:**
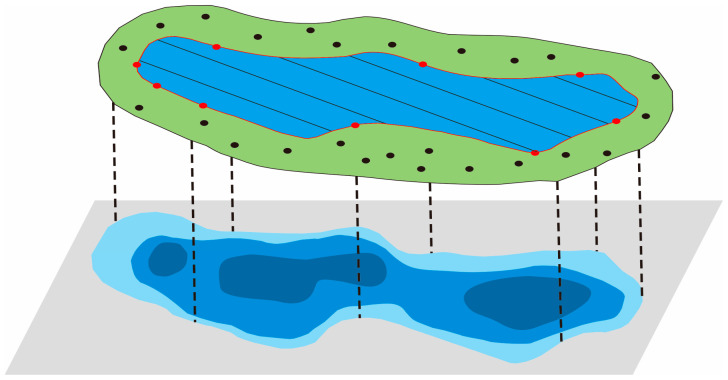
Schematic diagram of point cloud data fusion.

The topography of the coal mining subsidence area is mainly obtained through three methods: UAV LiDAR, unmanned survey ships, and GNSS-RTK. First, low-altitude drones are used to obtain point cloud data outside the water area, and then unmanned ships are used to obtain underwater measurement point cloud data within the water area. Finally, GNSS-RTK is used to supplement some measurement points at the junction of land and water and the DEM accuracy check points generated after the final point cloud was fused (Figure 6).

## 3. Monitoring of Coal Mining Subsidence Areas in Northern Anhui

### 3.1. Study Area

**Figure 7:**
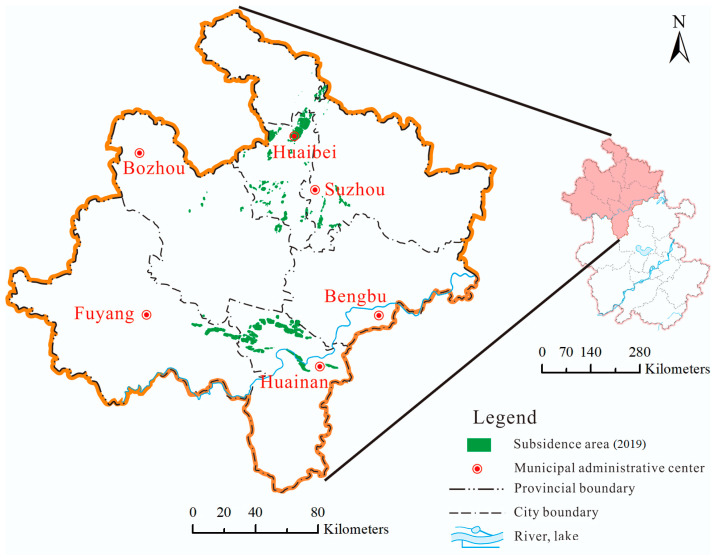
Location of coal mining subsidence area in northern Anhui.

The subsidence area is distributed in the Huainan mining area and the Huaibei mining area, covering 19 counties in six cities in northern Anhui (total area 22,569 km^2^), and belongs to the Huaibei Plain, Jianghuai Plain, and Huanghuaihai Economic Zone. The northern Anhui area is rich in groundwater. Due to different structural conditions, the water level gradually increases from south to north. It is a typical high water level mining area [36]. The landforms of the six cities in northern Anhui are mainly plains, with high levels in the west and low levels in the east. As of the end of 2022, the total coal reserves in the coal mining subsidence area of northern Anhui were 34.287 billion tons, making an important contribution to support the economic and social development of the Anhui province and even the Yangtze River Delta region, and effectively ensuring national energy security. With the large-scale mining in the Lianghuai mining areas (Huainan and Huaibei mining areas), large-scale surface subsidence has occurred, which has a great impact on the ecological environment and human life [37]. As of 2022, there are 121 coal mining subsidence areas with an area of 751 km^2^, and Huainan and Huaibei cities account for 76% of the coal mining subsidence areas (Figure 7).

### 3.2. Data Sets and Processing

In order to better carry out the investigation and monitoring of coal mining subsidence areas in northern Anhui, a large amount of data were collected from the Anhui Provincial Basic Surveying and Mapping Information Center, natural resource management departments, and coal mining enterprises responsible for the coal mining subsidence areas. Among them, the Sentinel-1A image data were downloaded through the European Space Agency (https://www.esa.int/, accessed on 6 May 2024). The main data are shown in Table 1.

### 3.3. Space–Air–Ground Collaborative Monitoring

Based on satellite remote sensing, UAV LiDAR, unmanned surveying ships and on-site field surveys, the coal mining subsidence area in northern Anhui is taken as the study area. A space–air–ground cooperative monitoring system for the coal mining subsidence area in northern Anhui was established, which obtains objective and true monitoring data and provides data services to the natural resource management department.

Different technical methods are used to obtain a large amount of monitoring data for mining subsidence areas. In order to ensure data consistency and facilitate data integration and analysis, ground control point data from the image control point library was collected from the Anhui Provincial Basic Surveying and Mapping Information Center. SAR image data were corrected into the CGSC2000 (China Geodetic Coordinate System 2000). For measurement technologies such as airborne LiDAR, unmanned measurement ships, and GNSS-RTK, the AHCORS (Anhui Continuous Operational Reference System) was connected during the measurement process to directly obtain data under the CGCS2000 coordinate system.

Using the leveling data in the mining area to check the InSAR monitoring data, the absolute difference in annual cumulative settlement values at each level point between the two is concentrated at [0, 4 cm], both of which are less than 5 cm.

For UAV LiDAR measurements, the digital elevation model grid scale is required to be 1 m, and the point cloud density should be greater than four per square meter. For unmanned surveying ships, in order to improve the accuracy of underwater terrain measurement as much as possible, measurements are carried out at intervals of 10 m between survey lines. For the 2 m × 2 m DEM data acquired by UAV LiDAR and unmanned surveying ships, GNSS RTK was used to measure the monitoring points on the spot. A total of 1573 detection points were measured, with a gross error rate of 1.8%, a maximum elevation accuracy error of 0.24 m, and a medium error of 0.09 m. The resulting data have passed the inspection of the Station of Surveillance and Examination for Surveying product in Anhui Province.

#### 3.3.1. Monitoring Changes of Subsidence Range

Based on the scope of coal mining subsidence areas in Anhui Province in 2019 issued by the Ministry of Natural Resources of China, InSAR technology was used to carry out surface subsidence and deformation monitoring in coal mining subsidence areas in northern Anhui from 2020 to 2022, combined with data reported by counties and cities and verification by outsiders. The scope of the subsidence area was investigated. Figure 6 shows the change process of the scope of the newly added subsidence area.

**Figure 8:**
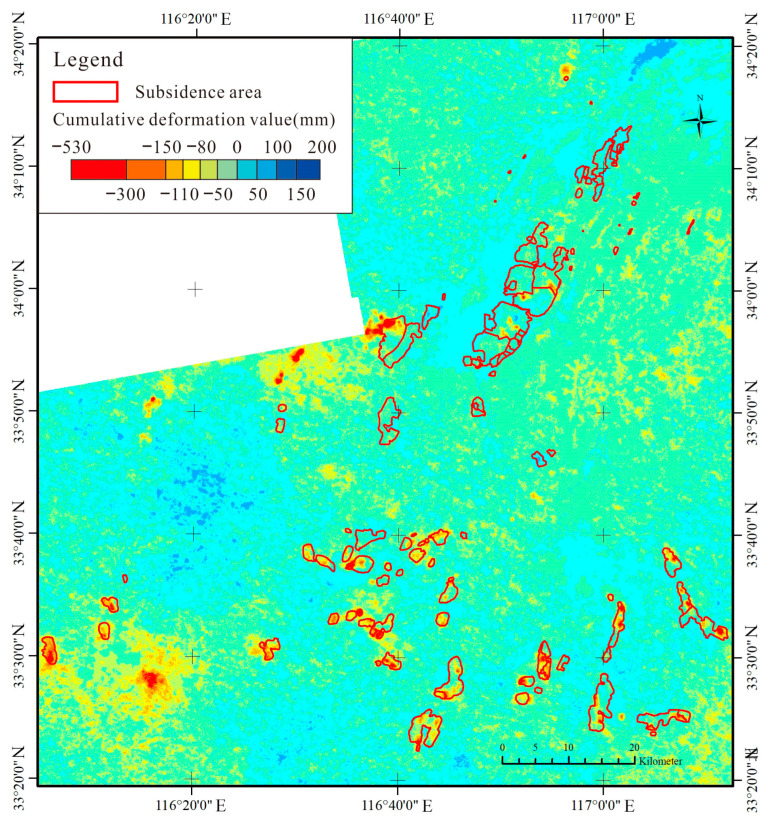
Cumulative deformation value of subsidence areas (Huaibei mining area).

**Figure 9:**
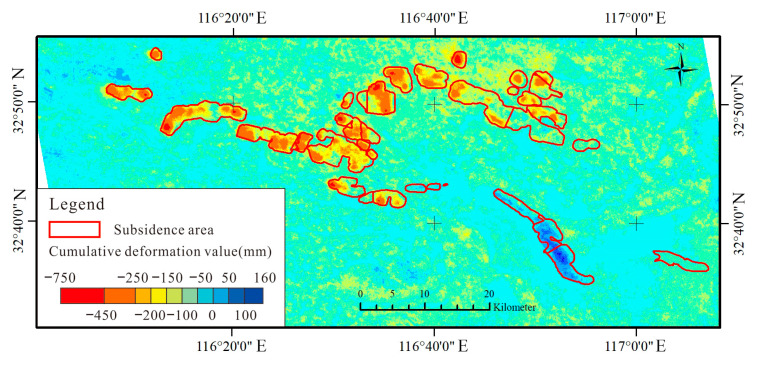
Cumulative deformation value of subsidence areas (Huainan mining area).

The paper mainly uses C-band Sentinel-1A data as the main SAR image source. Sentinel-1A was launched on 3 April 2014 as the Copernicus program carried out by the European Space Agency. The radar sensor carried by the satellite transmits in the C-band and has a revisit period of 12 days. We acquired Sentinel-1A data as the main SAR image source. According to the distribution of the subsidence area, a total of 119 groups of SAR images from 1 January 2020 to 31 December 2022, Frame101, and Frame96 in Path142 were selected. The professional processing software SARScape 5.6 was used, and SBAS-InSAR was used as the processing method to obtain the cumulative deformation amount of the study area in 2020, 2021, and 2022. The method to identify the boundary of coal mining subsidence area is shown as follows:

(1) In order to clarify the characteristics of the distribution and continuous expansion of subsidence areas, it is necessary to collect and organize images in the subsidence area in 2022, and create a satellite remote sensing image base map.
(2) The SBAS-InSAR processing method was used to obtain the surface deformation amount of the subsidence area in each month from 2020 to 2022, and then it was used to calculate the accumulated surface deformation amount and the maximum deformation amount of the subsidence pattern within the study time range (Figure 8 and Figure 9).
(3) In ArcGIS 10.2 software, the surface subsidence caused by coal mining in the study area was visually interpreted through human–computer interactions, and its subsidence edge range lines were outlined and represented as polygons.
(4) The boundary of coal mining subsidence was drawn by combining the coal mine subsidence range and spatial distribution map, mineral rights range map, and coal mining working surface map obtained through the above process.

#### 3.3.2. Monitoring Changes of Land Use in Coal Mining Subsidence Areas

To study the land use situation, it is necessary to obtain orthophoto data from 2012, 2017, and 2022. The image data for 2012 was from the images used in the second land survey in China. In 2017, 76 scenes of Gaofen-2 and 23 scenes of Beijing-2 were used. In 2022, 103 scenes of Zhonggaojing-1 and 224 scenes of Gaojing-2 were used. After orthorectification, registration, and mosaicking, digital orthophoto maps (2017 and 2022) were generated using pixel factory.

Combined with the data of the third land survey, according to the land use classification standards of the third land survey and the land type characteristics of the settlement area, it is divided into 9 land types, and the classification indicators are extracted, as shown in Table 2. Based on the land use survey data, the scope of the subsidence area in 2022 was matched, and the land use situation in the subsidence area was collected by manual visual interpretation. The ground subsidence characteristics of the study area are reflected in the water accumulation area formed by the subsidence. Priority should be given to extract elements such as lakes, pits, and ponds, and finally form a time series of monitoring vector results for coal mining subsidence areas [13]. Mainly the collection accuracy of classification boundaries of land use with obvious boundaries should be controlled within five pixels. In special cases, such as shadow areas, the collection accuracy should in principle be controlled within 10 pixels.

A total of 750 samples from the third land survey in 2012, 2017, and 2022 were collected, covering land use data in three periods, to verify the accuracy of the classification results. The overall classification accuracies in 2012, 2017, and 2022 were more than 92%.

#### 3.3.3. Monitoring of Subsidence Degree in Coal Mining Subsidence Areas

According to the “Anhui Province Coal Mining Subsidence Area Definition and Classification Standards”, coal mining subsidence areas with a subsidence value less than 500 mm are considered light subsidence areas, areas with a subsidence value between 500 mm and 1500 mm are considered medium subsidence areas, and areas with a subsidence value greater than 1500 mm are considered heavy subsidence areas [18].

Based on the 2 m × 2 m DEM data of the coal mining subsidence area in 2015, UAV LiDAR and unmanned surveying ships were used to obtain the current 2 m × 2 m DEM data, and the two were compared with each other [38]. The subsidence is calculated as follows:

(9) $$H_{i}=DEM_{i}^{2022}-DEM_{i}^{2015}$$

where $H_{i}$ represents the subsidence value of point i, $DEM_{i}^{2022}$ represents the elevation value of point i in 2022, and $DEM_{i}^{2015}$ represents the elevation value of point i in 2015.

**Figure 10:**
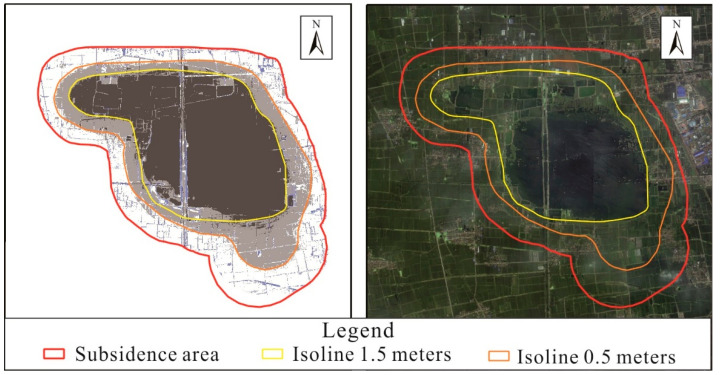
Distribution of subsidence depth in coal mining subsidence area.

After subtracting the two DEMs, the software ArcGIS 10.2 was used to classify them into 500 mm and 1500 mm, and superimpose the 2022 remote sensing orthophotos. The isoline data were extracted through human–computer interaction to obtain the range of different subsidence depths (Figure 10).

#### 3.3.4. Monitoring Scope of Coal Mining Subsidence Area Governance

In order to assist in the ecological restoration and management of coal mining subsidence areas and evaluate the actual governance effectiveness of subsidence control areas, the scope of coal mining subsidence governance areas and reclamation areas in northern Anhui is monitored using land survey data, high-resolution remote sensing images of spatial and temporal sequences, and industry thematic data.

The specific methods are as follows:

(1) The boundaries of the areas are collected under control by the end of 2022 from the Natural Resources and Planning Bureau of the prefecture-level cities involved, and the boundaries and governance status of the areas under control are sorted out.
(2) Based on remote sensing images and combining with the second and third land survey vector data, preliminary review of the governance area is conducted. For the uncertain scope of the governance area, on-site identification and signature confirmation methods are used for information processing, and the boundaries of the governance area are initially formed. Then, change statistics are used to find the latest land type changed to cultivated land and obtain the reclamation area.
(3) The results of subsidence area scope and other control project results in major projects in Anhui Province are integrated to form a more accurate boundary line of the governance area.
(4) The management department shall review the relatively complete boundary lines of the governance area and strictly control the project achievement data and governance effectiveness retained during the project governance process.

#### 3.3.5. Monitoring Scope of Stable Subsidence Areas in Coal Mining Subsidence Areas

The coal resources in northern Anhui are dominated by multi-coal seam structures. The coal seams are deeply buried, the mining cycle is long, and the multi-layer mining takes a long time to stabilize. In order to avoid duplication of investment in governance funds, the subsidence area governance should focus on being carried out in stable subsidence areas as much as possible. Therefore, the monitoring of stable subsidence areas is of great significance for the preparation of subsidence area governance plans.

According to the “Technical Regulations for Ecological Restoration of Mining Subsidence Areas”, coal mining subsidence areas where the surface point sinking value does not exceed 30 mm for 6 consecutive months are considered stable subsidence areas. Through Sentinel-1A data and SBAS-InSAR as a processing method, the 2022 mining subsidence area was obtained. The cumulative subsidence amount in the coal subsidence area was obtained in the past 6 months [39]. Then, combined with high-resolution remote sensing images, the range of stable subsidence areas was delineated.

## 4. Results and Analysis

### 4.1. Changes in the Scope of Subsidence Area for 2019–2022

Based on the basic situation survey of coal mining subsidence areas in Anhui Province in 2019, as of the end of 2022, there were 121 coal mining subsidence areas in six cities in northern Anhui, of which 16 were new, with a total area of 750.9 km^2^. From Figure 7, the total area of coal mining subsidence areas were increased by 8.1% when compared to 2019. The coal mining subsidence areas in descending order of area are as follows: Huainan, 292.5 km^2^ (39.0%), an increase of 11.5%; Huaibei, 279.54 km^2^ (37.2%), an increase of 2.7%; Suzhou, 74.3 km^2^ (9.9%), an increase of 3.9%; Fuyang, 65.9 km^2^ (8.8%), an increase of 15.8%; Bozhou, 32.8 km^2^ (4.4%), an increase of 23.3%; Bengbu, 5.9 km^2^ (0.7%), an increase of 20.4%.

**Figure 11:**
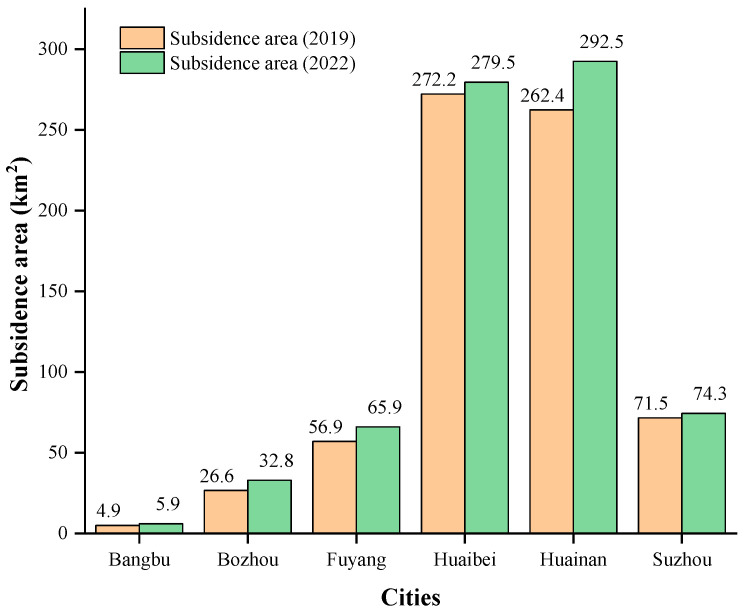
Changes in the area of coal mining subsidence from 2019 to 2022.

**Figure 12:**
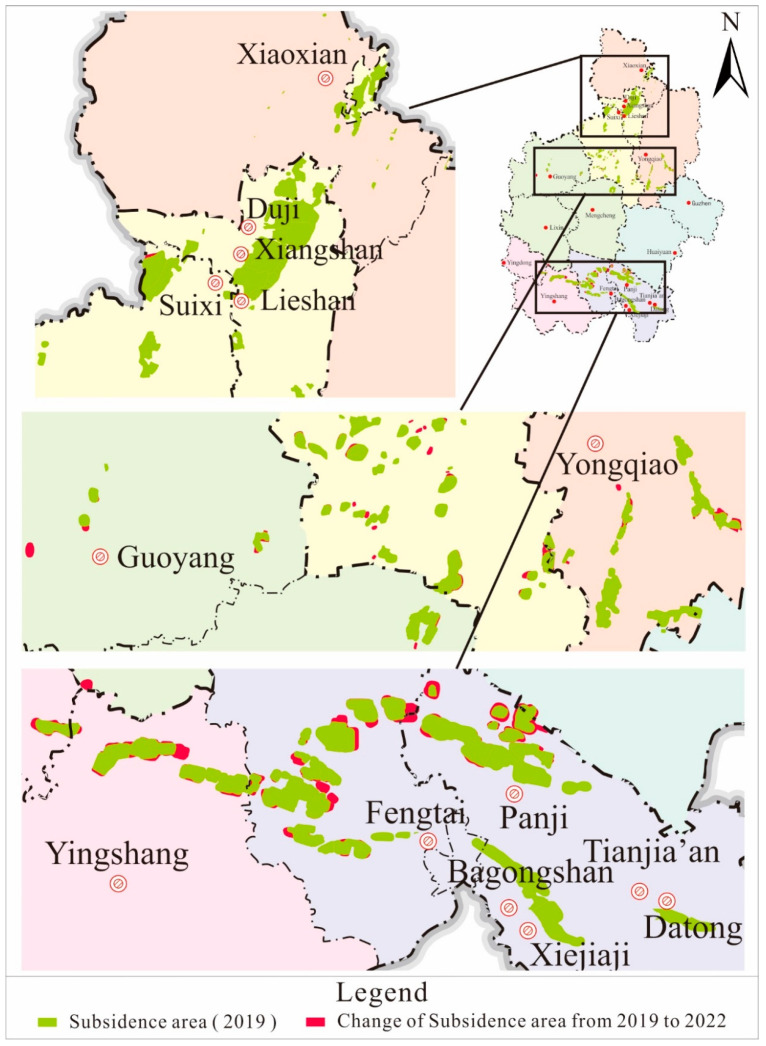
Changes in the distribution of coal mining subsidence areas (2019–2022).

From Figure 11 and Figure 12, the distribution of coal mining subsidence areas in 2022 is similar to that in 2019. It is mainly concentrated in the northern part of Huainan and the entirety of Huaibei, covering an area of 572.0 km^2^, accounting for approximately 76.2% of the total area of coal mining subsidence areas. Coal mining subsidence areas can be divided into the Huaibei mining area and the Huainan mining area, according to location distribution. The Huaibei mining area is mainly concentrated in Duji, Lieshan, Xiangshan, Suixi, Yongqiao, and Guoyang. The Huainan mining area is mainly concentrated in Fengtai, Panji, Bagongshan, and Yingshang. Since the recorded coal mining subsidence areas in 2019, Lixin and Bozhou have been added. Judging from the location of the increased subsidence areas, the subsidence range in the Huainan mining area has increased significantly, while that in the Huaibei mining area is relatively small.

### 4.2. Land Use Change from 2012 to 2022

As can be seen from Table 3, the coal mining subsidence area is mainly the cultivated land, water areas, and water conservancy facility land, which account for approximately 72% of the total area by 2022. Cultivated land is decreased from 383.8 km^2^ to 284.4 km^2^ at an average annual rate of 9.9 km^2^, while water areas are increased from 192.4 km^2^ to 294.3 km^2^ at an annual rate of 10.2 km^2^. Garden land, forest land, grassland, traffic and transportation land, and water conservancy facility land are also increased to varying degrees. The maximum increase was in grasslands, with an increase of 7.4 km^2^. The increase from large to small was as follows: water areas, grassland, forest land, traffic and transportation land, garden land, and water conservancy facility land. Residential land and industrial and mining land are decreased by 11.8 km^2^ and 9.6 km^2^, respectively. Garden land, industrial and mining land have a trend with first increasing and then decreasing between 2012 and 2022, while other land types show a continuous increase or decrease as a whole. For cultivated land and water areas, between the first 5 years and the next 5 years, the amount of change is relatively stable.

**Figure 13:**
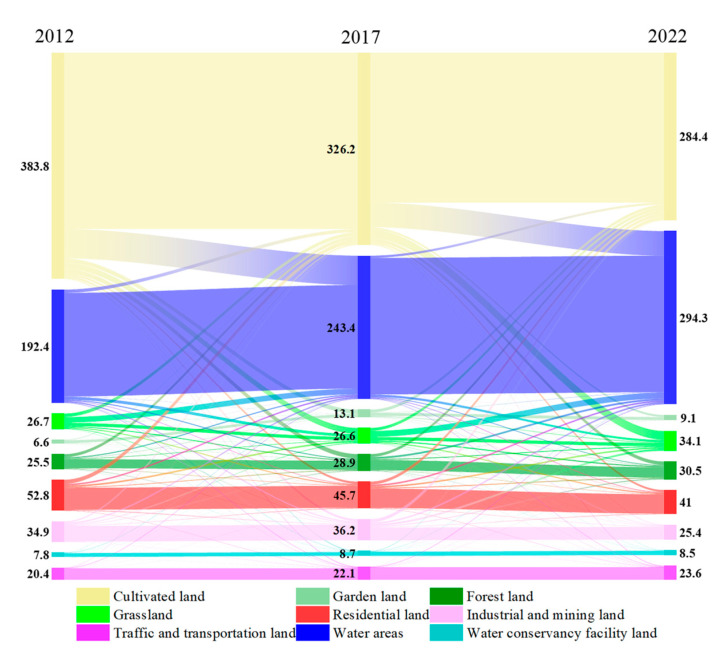
Land use transfer in coal mining subsidence areas from 2012 to 2022 (unit: km^2^).

From Figure 13, the loss of cultivated land is mainly converted into waters, and the inflow source of waters is mainly the cultivated land. The outflow area of water areas is very small, and nearly 50% of grassland is converted into waters. Residential land is continuously decreasing and mainly converted into the cultivated land. After 2017, the area of industrial and mining land converted into cultivated land is also increased significantly. The inflow and outflow of land for water conservancy facilities land and the land for transportation is very small and relatively stable.

### 4.3. Subsidence Degree of Coal Mining Subsidence Area

According to the difference between the two periods of 2 m × 2 m DEM and statistical analysis, the subsidence depth of the Huainan mining area is much greater than that of the Huaibei mining area. The maximum subsidence depth of the Huaibei mining area is generally from 1 to 5 m, and the maximum subsidence depth of the Huainan mining area is generally greater than 6 m. With respect to the changes in remote sensing images from 2015 to 2022, the coal mining subsidence area was initially divided into light subsidence areas, medium subsidence areas, and heavy subsidence areas.

According to the classification of the subsidence depth of the coal mining subsidence area, the subsidence depth of the coal mining subsidence area is shown in Table 4 below. The light subsidence area is 307.9 km^2^, the medium subsidence area is 161.8 km^2^, and the heavy subsidence area is 281.2 km^2^. The light, medium, and heavy subsidence areas in prefecture-level cities are shown in Table 4. Among them, the heavy subsidence area in Huainan is the largest, reaching 127.6 km^2^, accounting for 43.7% of Huainan’s coal mining subsidence areas and 45.4% of the total heavy subsidence areas; followed by Huaibei with 70.1 km^2^, accounting for 25.1% of Huaibei’s coal mining subsidence areas, and accounting for 24.9% of the total heavy subsidence areas. Fuyang and Bozhou have a larger proportion of heavy subsidence areas. The areas of heavy subsidence areas are 39.8 km^2^ and 14.0 km^2^, respectively, accounting for 60.3% and 43.6%.

### 4.4. Subsidence Area Governance and Reclamation Area Monitoring 

From Table 5 and Figure 11, the total area of the subsidence governance area is 199.2 km^2^, and the subsidence governance area accounts for 26.5% of the total subsidence area. From the perspective of cities, the governance rate of Huaibei reaches 51.3%, the governance rate of Huainan reaches 10.1%, and the governed rate of Fuyang reaches 13.6%.

As of 2022, the total reclamation area is 68.8 km^2^, accounting for 34.5% of the subsidence governance area. The six cities in northern Anhui Province in descending order of subsidence reclamation area are as follows: Huaibei, with 53.3 km^2^, Huainan, with 9.6 km^2^, Suzhou, with 3.3 km^2^, Bozhou, with 1.8 km^2^, Fuyang, with 0.7 km^2^, and Bengbu, with 0.1 km^2^.

At present, 276.1 km^2^ within the subsidence area has reached stable subsidence conditions, mainly distributed in the Huaibei mining area, accounting for about 60% of the total stable subsidence area. The stable areas from large to small are as follows: Huaibei, Huainan, Suzhou, Bozhou, Fuyang, and Bengbu. By superimposing the stabilizing area and the governance area, 99.2 km^2^ of the Huaibei governance area is within the stabilizing area, which is the largest area. The others, from largest to smallest are as follows: Huainan, Suzhou, Bozhou, Fuyang, and Bengbu. Huaibei and Suzhou both accounted for more than 60% of the governance areas, while Bozhou and Huainan accounted for 39% and 38%, respectively.

## 5. Discussion

### 5.1. Advantages of Space–Air–Ground Collaborative Monitoring in Coal Mining Areas

Data from space–air–ground collaborative monitoring are integrated and mutually verified to ensure accuracy, and technologies complement each other to ensure all-round efficiency. Satellite remote sensing can monitor large-scale areas with providing macro-decision making for natural resource management [40]. Low-altitude UAVs, unmanned survey ships, and field observations can provide the refined monitoring.

UAV is flexible to collect remote sensing data and can timely and effectively monitor the surface conditions of coal mining subsidence areas with producing high-precision DEM data. For water accumulation areas in subsidence areas, UAV LiDAR cannot measure it. Unmanned surveying ships and traditional measurement equipment can be used to produce the underwater topography of the water accumulation areas. After the two parts of data are fused, a complete high-precision DEM of the subsidence area is formed.

When InSAR technology monitors large gradient deformation in subsidence areas, the accuracy and reliability are difficult to guarantee [41]. For the subsidence changes in subsidence areas from 2015 to 2022, high-precision DEM data analysis is more accurate and can accurately obtain the range of different subsidence depths in subsidence areas. However, obtaining high-precision DEM through UAV and unmanned surveying ships is costly and inefficient. Therefore, in view of the short-term elevation changes in the subsidence area, InSAR has higher efficiency and lower costs [11].

### 5.2. Causes for Changes in the Scope of Subsidence Areas

The Lianghuai mining area in Anhui Province is an important coal production base in China, with a coal-containing area of 18,000 square kilometers. It is one of the 14 national large-scale coal bases with a capacity of 100 million tons and one of six coal-fired power bases identified in the “National Large Coal Base Construction Plan”. Therefore, coal mining subsidence areas are mainly concentrated in Huainan and Huaibei regions, with relatively few in the other four cities, accounting for only about 24%. Judging from the expansion of the subsidence area from 2019 to 2022, Huainan has grown by 11%, which is about four times the growth rate of Huaibei. The reason is that in recent years, in the context of urban transformation and overcapacity reduction, Huaibei has closed many coal mines and so economic growth was slowed down. Huainan is accelerating the transformation and upgrading of traditional industries, with the goal of refining and optimizing the coal power industry and turning pillar industries into advantageous industries, and is striving to build a USD 100 billion coal, electricity, chemical gas industry chain and lay a solid foundation for Huainan’s transformation. Huainan has large coal reserves, so most coal mines are still being mined, and the subsidence area is also expanding. It also explains that the Huainan mining area, which is affiliated to Fuyang, Bengbu, and Huainan, has a significantly higher subsidence area growth than the Huaibei mining area. The Huaibei Mining Area mainly includes Suzhou, Bozhou, and Huaibei.

### 5.3. Causes of Land Use Changes in Subsidence Areas

The cultivated land area of the six cities in northern Anhui accounts for nearly 50% of Anhui Province and belongs to the Huang-Huai-Hai Plain. Agriculture has always been a pillar industry in the region and an important grain and cotton production base in China. With coal mining, surface subsidence has formed large areas of water. As of 2022, the severe subsidence area is approximately 281.2 km^2^, most of which is water. In the end, the main land types within the coal mining subsidence area are cultivated land, water areas, and land for water conservancy facilities, accounting for approximately 72% of the total area. Land types such as gardens, forests, grasslands, and construction land are relatively small, accounting for only 28%. During the coal mining process, a series of basic service facilities have been formed with the occupancy of coal companies. There are relatively large amounts of construction land mainly used for the residences of the coal miners, industrial and mining land formed by coal mining, and transportation land required for coal transportation, accounting for 50% of the land area other than cultivated land, water areas, and water conservancy facilities land.

**Figure 14:**
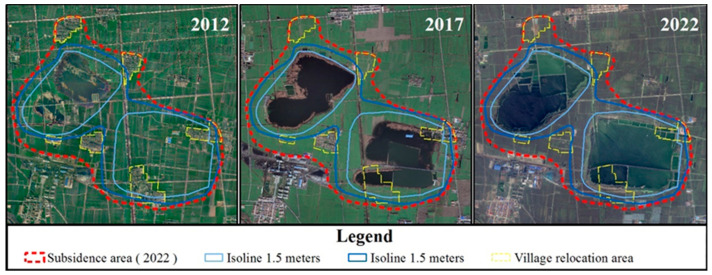
Typical characteristics of land use changes in coal mining subsidence areas.

From the perspective of time changes, we can directly see from Figure 14 that the reduction in cultivated land has a strong positive correlation with the increase in water areas and water conservancy facilities. With the surface subsidence, a large amount of cultivated land was destroyed and turned into water areas. From the analysis of three periods of data in 2012, 2017, and 2022, the rate of reduction in cultivated land and the rate of increase in water areas and water conservancy facilities land have slowed down to a certain extent since 2017. This is related to policies such as economic transformation and energy model conversion. Most coal mines in the Huaibei region have closed their pits, and the coal mining volume in the Huainan region is also decreasing. In addition, the country’s green transformation around resource depletion and increased reclamation tasks in coal mining subsidence areas have also decreased the loss of cultivated land [42,43].

In addition to changes in cultivated land, water areas, and water conservancy facilities, changes in other land types are also closely related to the governance of subsidence areas. Village relocation and management have caused a reduction in construction land, coal mine closures, industrial and mining abandoned land reclamation, etc., all of which have caused a reduction in industrial and mining land. Suitable management model of “planting trees on forest land” will affect the increase in forest land.

### 5.4. Suggestions for Subsidence Area Management

The total governance area of the subsidence area is 191.93 km^2^, accounting for 25.33% of the total subsidence area. The governance rate is not high, and the coal mining subsidence area has an expansion trend. The relocation and comprehensive management of villages in coal mining subsidence areas will be a long-term, arduous, and complex task. The scope of new subsidence areas from 2019 to 2022 is mainly cultivated land, accounting for 69% of the total newly added subsidence areas, which is much higher than the proportion of cultivated land in subsidence areas (38.1%). Therefore, as far as the newly added subsidence areas are concerned, coal mining subsidence will pose a greater threat to cultivated land.

In order to avoid duplication of investment in subsidence area governance, we should mainly consider carrying out governance and reclamation within the scope of stable subsidence areas [44]. From the perspective of actual governance, only Huaibei and Suzhou have stable subsidence area ratios with exceeding 60% in the governance areas, and other areas are relatively low. The main reason is that on the one hand, Huaibei and Suzhou coal mines have more closed pits and the areas of stability are larger; on the other hand, before the implementation of the governance, the management departments of Huaibei and Suzhou considered the scope of stability to a certain extent, so that they can be accurately implement repairs. The stabilized area in the treatment area only accounts for 43.68% of the total stabilized area, showing that the governance and reclamation process is slow.

Through the above analysis, the expansion of the coal mining subsidence area scope and the addition of new subsidence locations may have a huge impact on people’s lives. Efforts should be made to conduct regular monitoring of the location and scope of coal mining subsidence areas in northern Anhui and promptly discover new subsidence locations. In addition, the depth of subsidence must be predicted in advance so that appropriate methods can be adopted for subsidence governance and reclamation for risk avoidance. These issues should be repeated in the governance of the coal mining subsidence area management in order to increase the stability monitoring of coal mining subsidence, and to choose the appropriate time to carry out comprehensive governance.

### 5.5. Future Study and Prospects

This paper uses satellite remote sensing, UAV LiDAR, unmanned survey ships, GNSS-RTK, and field measurement surveys data to build a space–air–ground integrated collaborative monitoring system for coal mining subsidence areas. The surface subsidence changes, subsidence depth classification, land use spatiotemporal changes, water accumulation evolution in subsidence areas, and restoration and management monitoring were studied.

For the first time, from the perspective of natural resource management needs and with a focus on multiple business departments such as cultivated land resource protection, land space planning, land space ecological restoration, and natural resource survey, the changes in the coal mining subsidence area in northern Anhui and the causes were systematically studied. The results provide suggestions for various departments from a scientific perspective and data products to support natural resource management.

The formation and evolution of coal mining subsidence areas are closely related to underground mining activities [45]. This article uses remote sensing satellites, airborne LiDAR, and other techniques to monitor the surface characteristics in the subsidence area, which remains limited to the external manifestations on the ground. It is necessary to further collect underground information such as the amount of coal mined, the location of the mining working face, and the mining working time, and analyze the internal causes and evolution possibilities of subsidence in a higher dimension in the future.

Furthermore, in order to better serve natural resource management, monitoring of subsidence areas is of great significance. Identifying the scope of subsidence areas, stable subsidence areas, and governed areas all provide effective data support for scientific governance of subsidence areas. Moreover, other techniques or data should be used for more detailed subsidence monitoring, e.g., GNSS-Reflectometry, satellite gravimetry, etc. [46,47,48,49,50,51,52]. Furthermore, the next step should collect more historical and current data and establish an artificial intelligent model to understand and predict changes in future subsidence areas.

## 6. Conclusions

Based on the needs of comprehensive management of coal mining subsidence areas in northern Anhui, a space–air–ground collaborative monitoring system is constructed for the coal mining subsidence area in northern Anhui. The subsidence characteristics and its causes of coaling areas are investigated in North Anhui, including surface deformation, land use changes, subsidence change monitoring, and governance in the subsidence area. The main results of this study are summarized in the following:

(1) As of the end of 2022, 121 coal mining subsidence areas are found in six cities in northern Anhui, with 16 new ones and a total area of 750.9 km^2^. Compared with 2019, the total area of coal mining subsidence areas is increased by 8.14%. The coal mining subsidence areas in descending order of area are as follows: Huainan (292.5 km^2^), Huaibei (279.5 km^2^), Suzhou (74.3 km^2^), Fuyang (65.9 km^2^), Bozhou (32.8 km^2^), and Bengbu (5.9 km^2^).
(2) The main land types of coal mining subsidence areas in 2022 are waters areas and cultivated land, with 294.3 km^2^ and 284.4 km^2^, respectively. Other land types from large to small are as follows: residential land, grassland, forest land, industrial and mining land, traffic and transportation land, garden land, and water conservation facility land.
(3) From 2012 to 2022, the land area decreases from large to small are as follows: cultivated land, residential land, and industrial and mining land. The increases in area of land types from large to small are as follows: water areas, grassland, forest land, traffic and transportation land, garden land, and water conservancy facility land.
(4) Within the coal mining subsidence area, the light subsidence area is 307.9 km^2^, the medium subsidence area is 161.8 km^2^, and the heavy subsidence area is 281.2 km^2^. The area of light subsidence area and the area of medium subsidence area from largest to smallest are as follows: Huaibei, Huainan, Suzhou, Fuyang, Bozhou, and Bengbu. The areas of heavy subsidence areas from large to small are as follows: Huainan, Huaibei, Fuyang, Suzhou, Bozhou, and Bengbu.
(5) As of 2022, the total area of coal mining subsidence governance area is 199.2 km^2^, the total reclamation area is 68.8 km^2^, and the area of stable area is 276.1 km^2^. The areas of governance areas from large to small are as follows: Huaibei, Huainan, Suzhou, Fuyang, Bozhou, and Bengbu. The reclamation area areas from largest to smallest are as follows: Huaibei, Huainan, Suzhou, Bozhou, Fuyang, and Bengbu. The areas of stable areas from large to small are as follows: Huaibei, Huainan, Suzhou, Bozhou, Fuyang, and Bengbu.

## Figures and Tables

**Table 1:** The used data type and data sources.

Data Name	Data Content and Specifications
Digital Orthophoto data	High-resolution satellite images in 2012, 2017, and 2022 (better than 2 m).
Digital elevation model	Digital elevation model (2015), 2 m × 2 m (vertical accuracy in plain areas is less than 0.15 m).
Land use and land cover data	Anhui Province Land Survey Data (2012, 2017, 2022).
Coal mining subsidence area information	Coal mining subsidence area vector data in 2019, coal mining subsidence area statistical report results in 2022.
SAR remote sensing data	Sentinel-1A data from 2020 to 2022, resolution 5 m × 20 m.
Industry thematic information	Land remediation project data from 2019 to 2022, coal mining subsidence area situation table, etc.

**Table 2:** Land use classification standards.

Land Use Type	Description
Cultivated land	Crops are mainly grown in the surface cultivation layer, and land is planted for one season or more every year.
Garden land	Perennial woody and herbaceous crops that are intensively managed mainly for collecting fruits, leaves, roots, stems, juices, etc., including land used for nursery.
Forest land	Land where trees, bamboos, and shrubs grow.
Grassland	Land growing mainly herbaceous plants.
Residential land	The land with buildings and structures used for urban and rural residences and public facilities.
Industrial and mining land	Land mainly used for industrial, mining, and other production.
Traffic and transportation land	Land used for surface lines, stations, etc., for transportation.
Water areas	Land waters, ditches, lake surfaces, etc.
Water conservancy facility land	Land for hydraulic structures and other areas.

**Table 3:** Land use changes in coal mining subsidence areas from 2012 to 2022 (unit: km^2^).

Land Use Type	2012	2017	2022	Changes (2012–2022)
Cultivated land	383.8	326.2	284.4	−99.3
Garden land	6.6	13.1	9.1	2.6
Forest land	25.5	28.9	30.5	5.0
Grassland	26.7	26.6	34.1	7.4
Residential land	52.8	45.7	41.0	−11.8
Industrial and mining land	34.9	36.2	25.4	−9.6
Traffic and transportation land	20.4	22.1	23.6	3.2
Water areas	192.4	243.4	294.3	101.9
Water conservancy facility land	7.82	8.71	8.5	0.68

**Table 4:** Areas of light, medium, and heavy subsidence in northern Anhui.

Cities	Area (km^2^)		
	Light Subsidence Area	Medium Subsidence Area	Heavy Subsidence Area
Bengbu	2.3	1.5	2.2
Bozhou	9.1	9.8	14.0
Fuyang	13.8	12.3	39.8
Huaibei	146.7	62.8	70.1
Huainan	106.2	58.5	127.6
Suzhou	29.8	16.9	27.5
Total	307.9	161.8	281.2

**Table 5:** The status of coal mining subsidence area governed in 2022 (unit: km^2^).

Cities	Subsidence Governed Area	Reclamation Area	Stable Subsidence Area	Stable Area in the Governed Area	Reclamation Area/Subsidence Governed Area
Bengbu	0.4	0.1	0.8	0.1	17.5%
Bozhou	3.6	1.8	4.4	1.4	50.0%
Fuyang	8.9	0.7	1.3	0.2	7.5%
Huaibei	143.4	53.3	165.2	99.2	37.1%
Huainan	29.4	9.6	54.3	11.1	32.7%
Suzhou	13.5	3.3	50.1	8.7	24.3%
Total	199.2	68.8	276.1	120.7	34.5%

## Data Availability

Data are available on request due to restrictions. The data presented in this study are available on request from the lead author, upon reasonable request.
